# Supplementary material for: Atomic layer deposition triggered Fe-In-S cluster and gradient energy band in ZnInS photoanode for improved oxygen evolution reaction
Source: Nat Commun. 2021 Sep 2;12:5247. doi: 10.1038/s41467-021-25609-0 (PMC8413305; doi:10.1038/s41467-021-25609-0)
Supplement: Supplementary file 1 — Supplementary Information [file 41467_2021_25609_MOESM1_ESM.pdf]

Supporting Information

Atomic layer deposition triggered Fe-In-S cluster and gradient energy band in ZnInS photoanode for improved oxygen evolution reaction

Meng et al.

Supplementary Figures

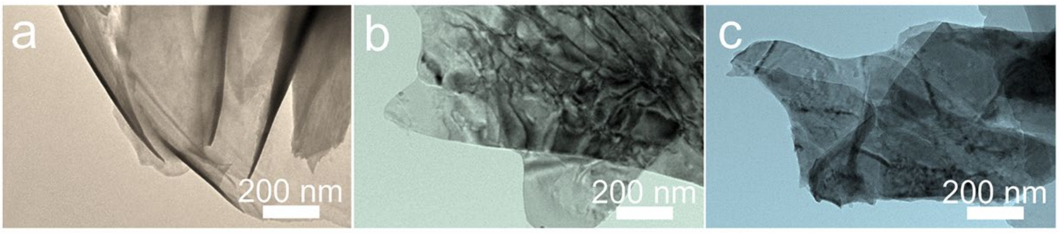

Supplementary Figure 1. TEM images. TEM images of (**a**) ZIS, (**b**) ZISZ, and (**c**) ZISZ/Fe nanosheets.

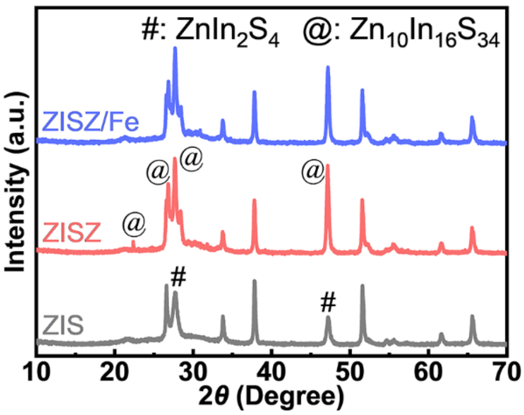

Supplementary Figure 2. XRD pattern of ZIS, ZISZ, and ZISZ/Fe.

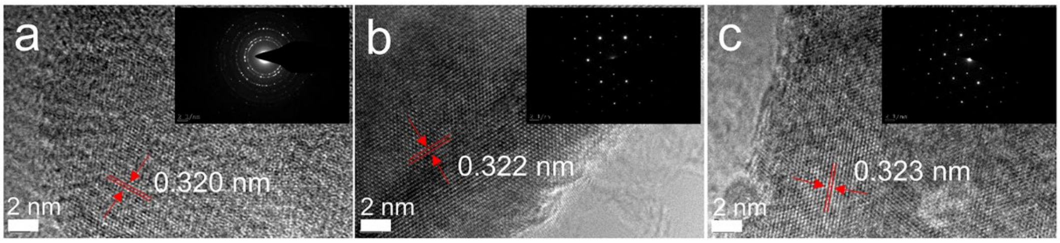

Supplementary Figure 3. TEM images. HRTEM images and corresponding SAED patterns of (**a**) ZIS, (**b**) ZISZ, and (**c**) ZISZ/Fe nanosheets.

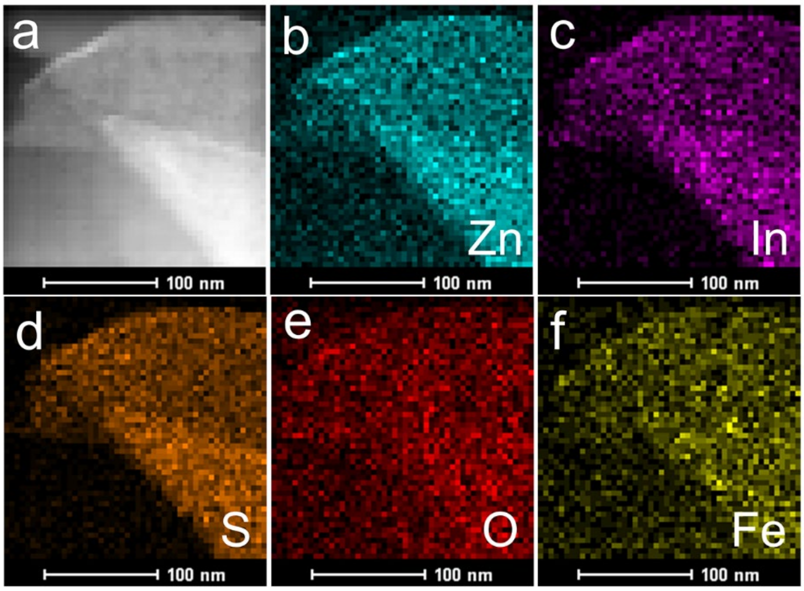

Supplementary Figure 4. The elemental mapping of ZISZ/Fe.

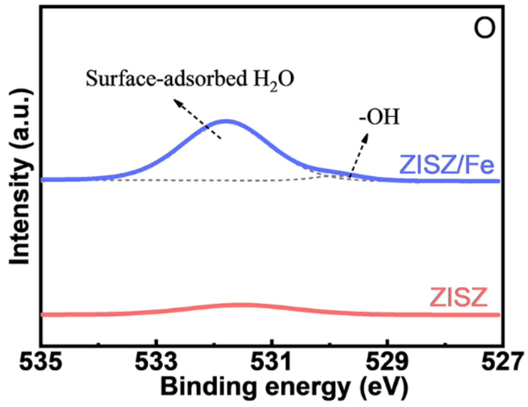

Supplementary Figure 5. XPS spectra for O 1*s*. The O 1*s* XPS spectra of ZISZ and ZISZ/Fe nanosheets.

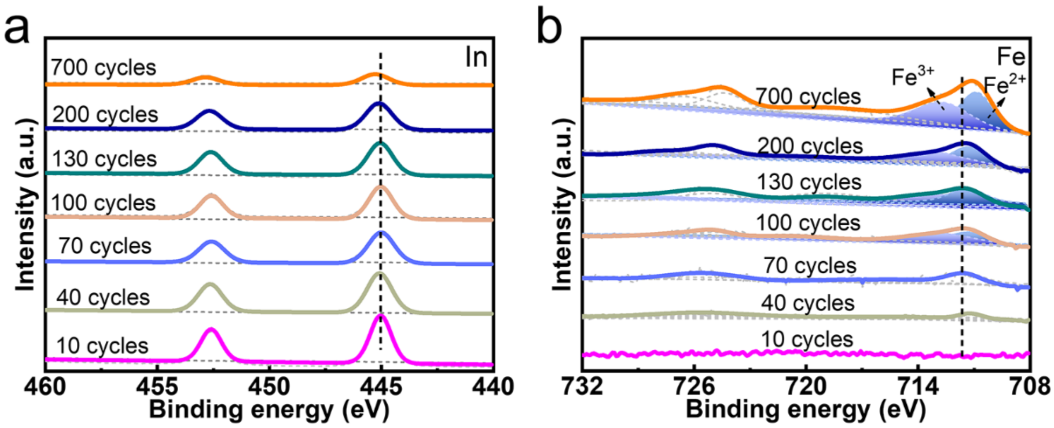

Supplementary Figure 6. XPS spectra for In 3*d* and Fe 2*p*. XPS spectra of (**a**) In 3*d* and (**b**) Fe 2*p* of ZISZ with different ALD cycles.

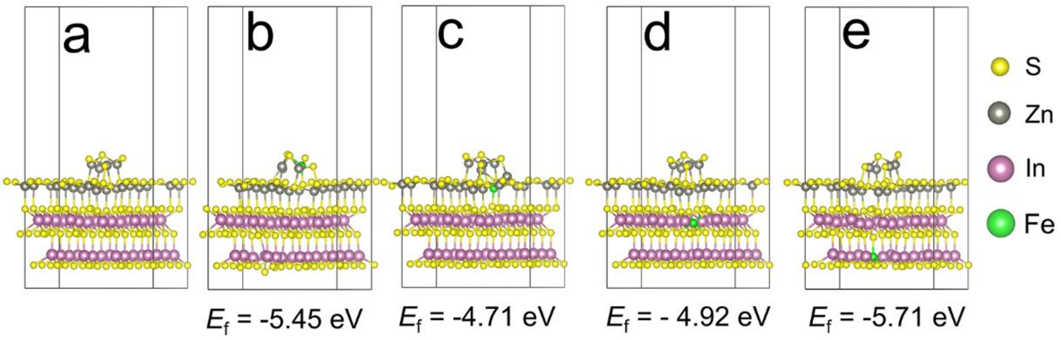

Supplementary Figure 7. **Models for formation energy calculations.** Simulation cell showing the optimized geometries of (**a**) pristine ZISZ (001) surface and ZISZ/Fe structures of Fe substituting to (**b, c**) Zn and (**d, e**) In. The formation energies (E_f) listed below each geometry (b-e) are obtained using the formula: $E_f = E_{ZISZ/Fe} - E_{ZISZ} - E_{Fe} + E_{Zn(In)}$. Here, $E_{ZISZ/Fe}$ and E_{ZISZ} are the total energy of the ZISZ/Fe and ZISZ systems, and E_{Fe} and $E_{Zn(In)}$ correspond to the energy of a single Fe, and Zn or In atom, calculated using the unit cell of each bulk material. Greater negative value of formation energy means easier substitution. Correspondingly, the estimation of OER efficiencies between pristine ZISZ and pristine ZISZ doping with Fe are determined by the systems (**a**) and (**e**).

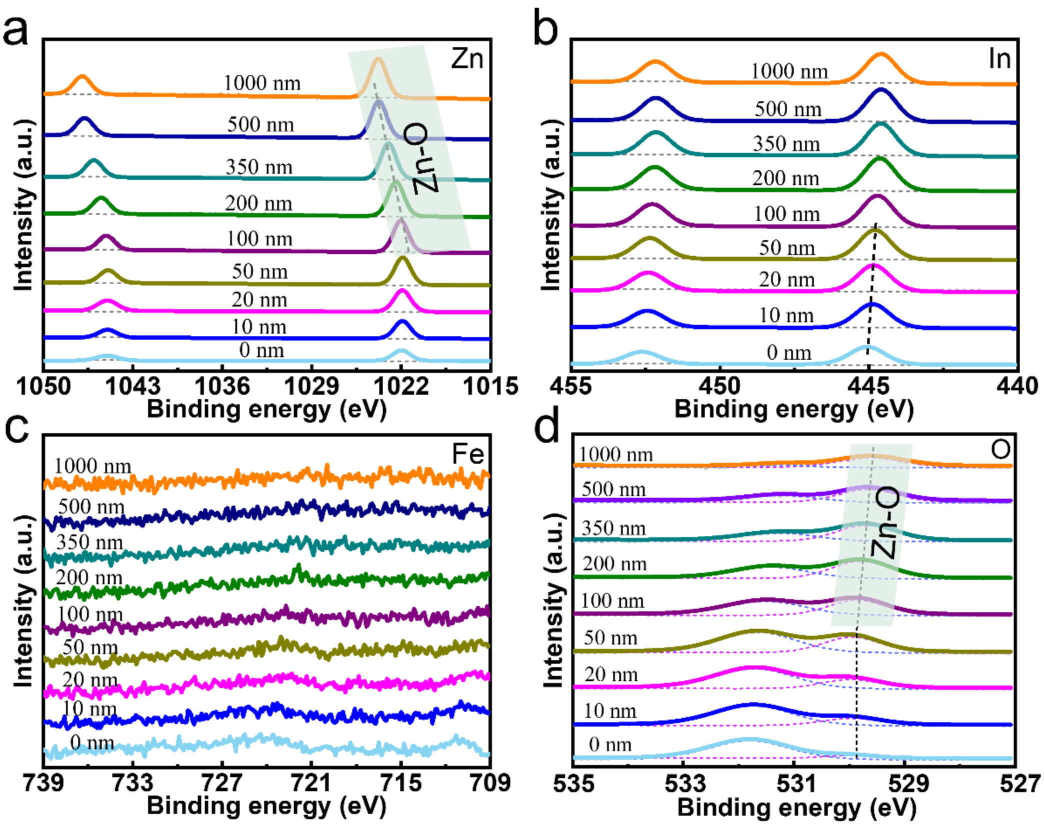

Supplementary Figure 8. XPS spectra to illustrate the states of photoanodes. XPS spectra from ZISZ/Fe at different etching depth. (**a**) Zn 2*p*, (**b**) In 3*d*, (**c**) Fe 2*p*, and (**d**) O 1*s*.

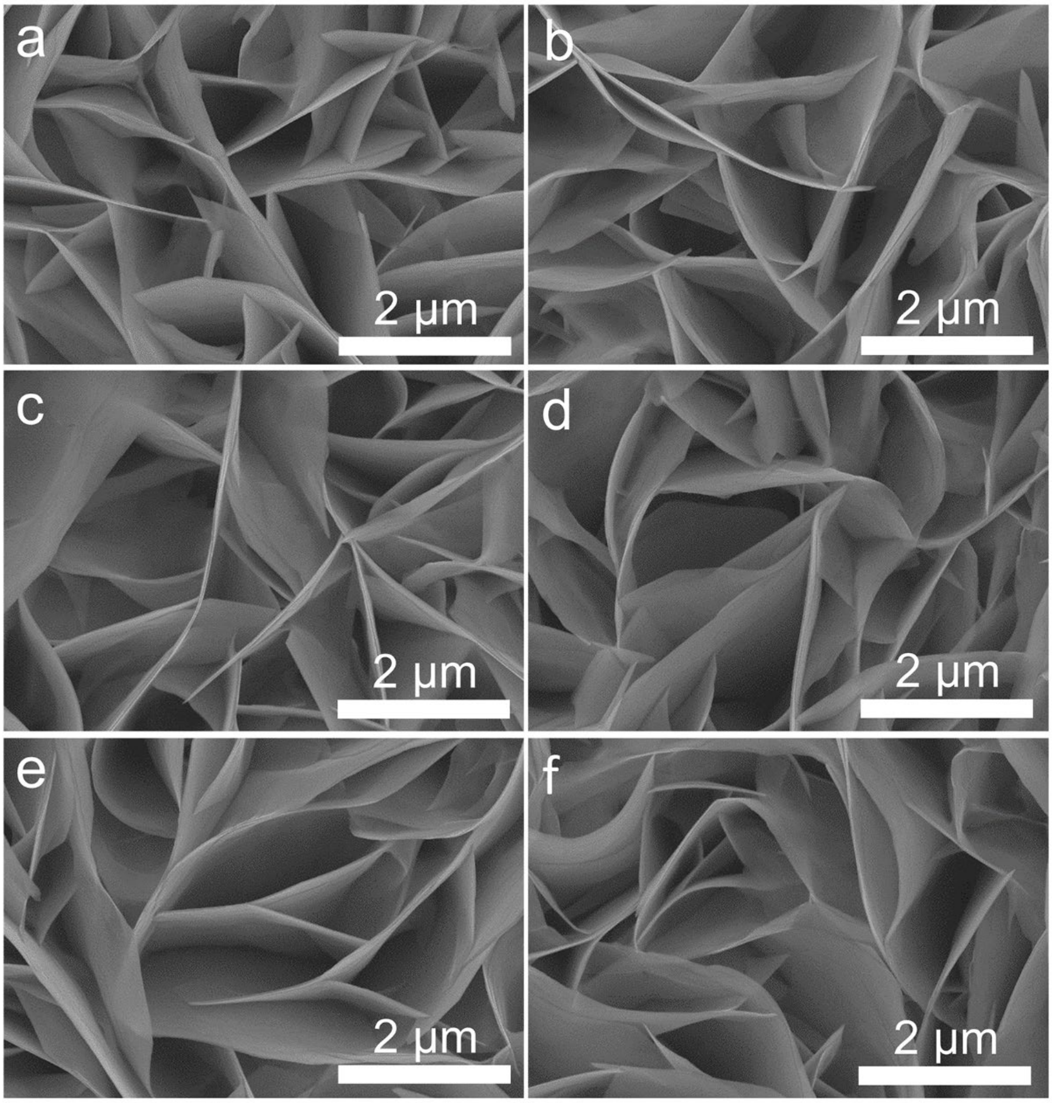

Supplementary Figure 9. SEM images of ZISZ/Fe with different ALD cycles. (**a**) 10 cycles, (**b**) 40 cycles, (**c**) 70 cycles, (**d**) 100 cycles, (**e**) 130 cycles, and (**f**) 700 cycles.

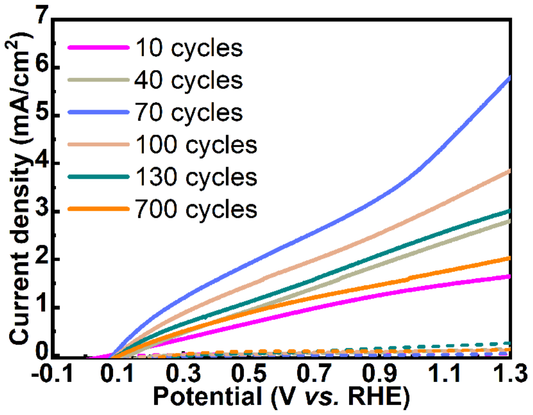

Supplementary Figure 10. ***J-V*** **curves.** Linear sweep voltammogram curves of ZISZ with different ALD cycles measured in 0.5 M Na_2SO_4.

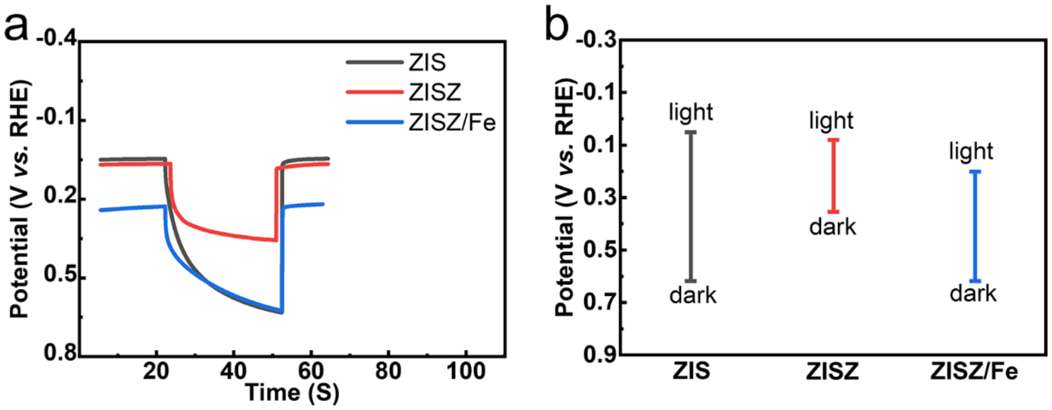

Supplementary Figure 11. The photovoltage positions. (**a**) OCP measurements under dark and light conditions and (**b**) OCP values of ZIS, ZISZ, and ZISZ/Fe photoanodes, respectively.

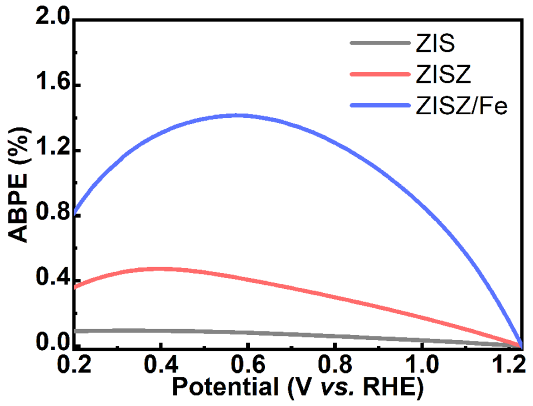

Supplementary Figure 12. The η_{ABPE} curves of ZIS, ZISZ, and ZISZ/Fe.

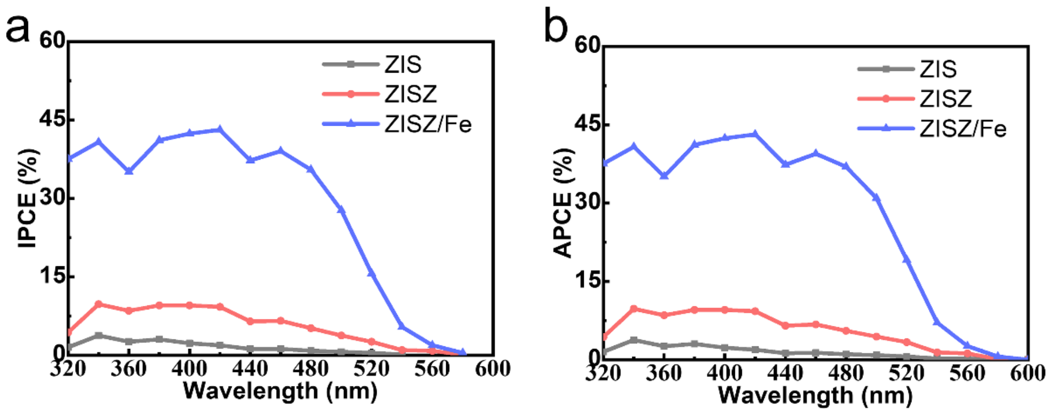

Supplementary Figure 13. (**a**) IPCE curves and (**b**) APCE values of ZIS, ZISZ, and ZISZ/Fe photoanodes, respectively.

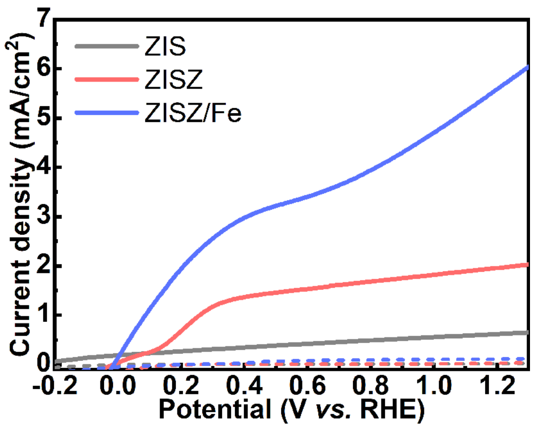

Supplementary Figure 14. *J-V* curves. Linear sweep voltammogram curves of ZIS, ZISZ, and ZISZ/Fe. 0.25 M Na_2SO_3 + 0.25 M Na_2S are used as hole scavenger.

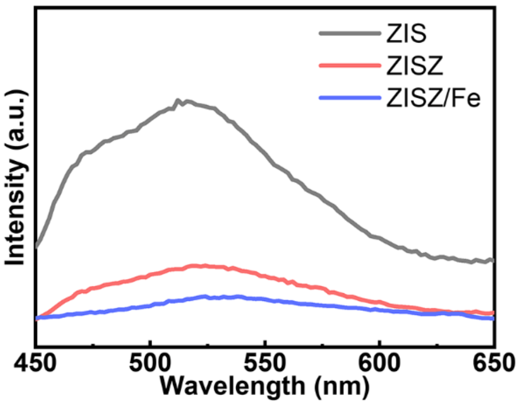

Supplementary Figure 15. The PL spectra of ZIS, ZISZ, and ZISZ/Fe.

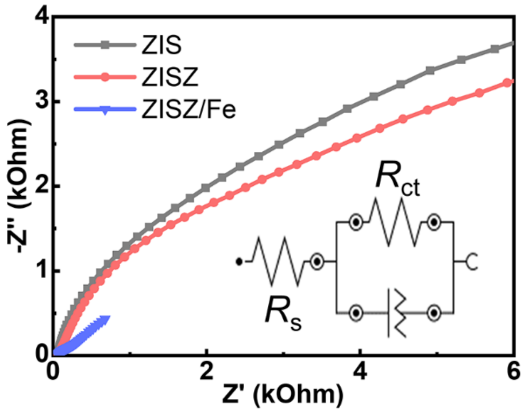

Supplementary Figure 16. EIS Nyquist plots of ZIS, ZISZ, and ZISZ/Fe (the inset is the fitting circuit diagram).

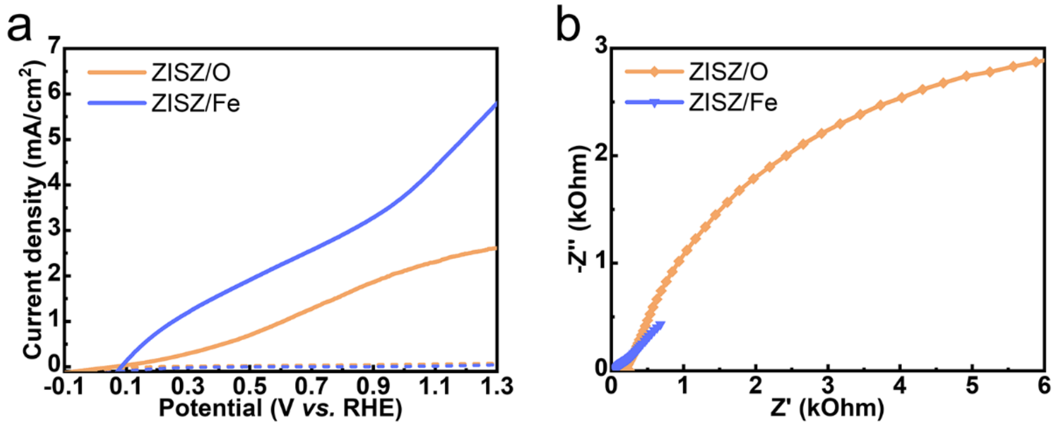

Supplementary Figure 17. Experiment comparison. (**a**) Linear sweep voltammogram curves, (**b**) EIS plots of ZISZ/O (only O_3) and ZISZ/Fe.

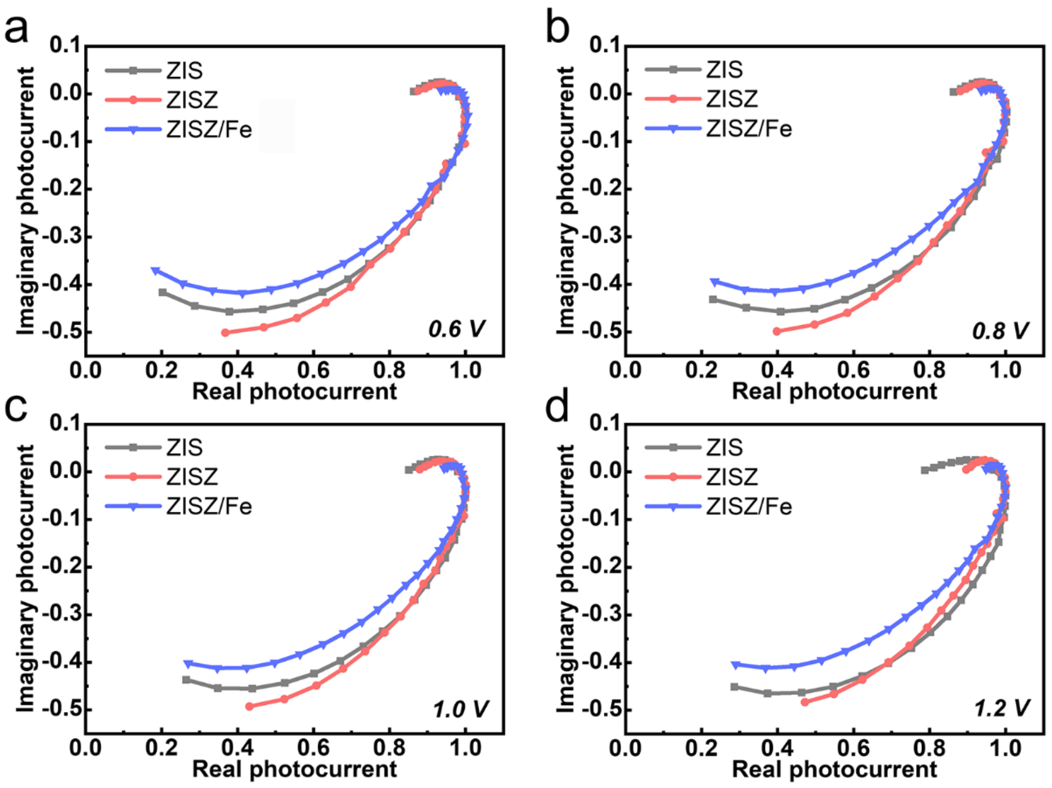

Supplementary Figure 18. The IMPS results. IMPS plots of ZIS, ZISZ, and ZISZ/Fe at different potential. (**a**) 0.6 V, (**b**) 0.8 V, (**c**) 1.0 V, and (**d**) 1.2 V.

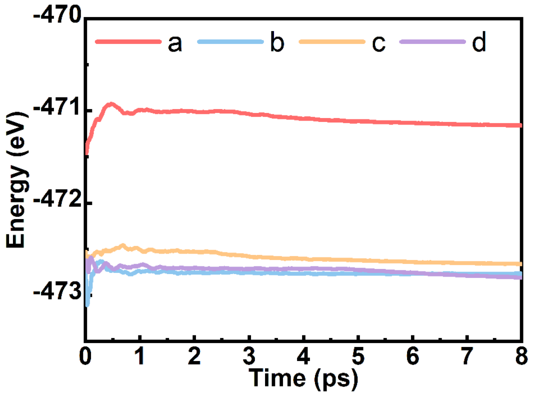

Supplementary Figure 19. Evolution of total energy cumulative average over time for four systems oscillates only in a narrow window. Evolution of total energy of the 8 ps adiabatic MD trajectories in the microcanonical ensemble of pristine ZISZ (**a**) and (**b-d**) ZISZ/Fe systems. Small energy fluctuations indicate that the four systems used in the present work are thermodynamically stale.

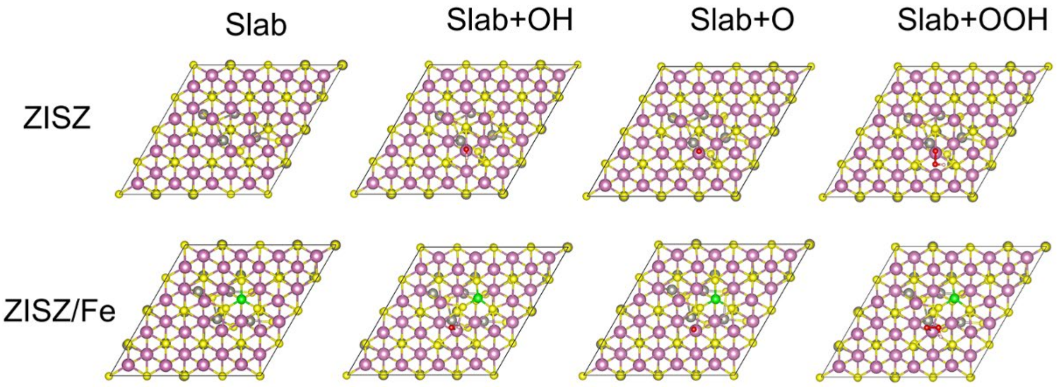

Supplementary Figure 20. All the optimized structures of the OER four-step reaction for both the ZISZ and ZISZ/Fe systems.

Supplementary Tables

Supplementary Table 1. The *J* of ZISZ/Fe in comparison with other sulfide based photoanodes.

Photoelectrode materials	Electrolyte	*J* @ 1.23 V *vs.* RHE (mA cm^{-2})	References
V_s-$CdIn_2S_4$	Na_2SO_4	4.76	1
$ZnIn_2S_4/TiO_2$	Na_2S/Na_2SO_3	1.75	2
In_2S_3/Bi_2S_3	Na_2SO_4	2.0	3
WO_3/In_2S_3	Na_2SO_4	1.61	4
In_2S_3/In_2O_{3-x}	Na_2SO_4	1.28	5
$ZnIn_2S_4/TiO_2/SiO_2$	Na_2S/Na_2SO_3	0.80	6
$ZnIn_2S_4$/CoPi	Na_2S/Na_2SO_3	0.90	7
ZnO/ZnS	Na_2SO_4	1.3	8
CdS Nanorod/ hexagonal SnS_2	Na_2SO_4	1.59	9
$ZnIn_2S_4$/RGO/ZnO	Na_2SO_4	2.30	10
Vertical SnS_x/CdS	Na_2SO_4	2.6	11
Vertical SnS_x/CdS	Na_2SO_3	4.5	11
SnS_2/SnS/OS	Na_2SO_4	2.15	12
ZnS/CdS	Na_2S/Na_2SO_3	5.1	13
CdS/ZnO/$ZnFe_2O_4$	Na_2S	4.2	14
TiO_2/CdS/MoS_2	Na_2S/Na_2SO_3	3.25	15
ZnInS/Fe-In-S	Na_2SO_4	5.35	This work

Supplementary Table 2. The free energy changes of four elementary steps for OER in ZISZ, and ZISZ/Fe systems when the applied potential is 0 V or 1.23 V, and the unit of free energy is eV.

	U (V)	step1	step2	step3	step4
ZISZ	0	0.536	0.047	3.522	0.815
	1.23	-0.694	-1.183	2.292	-0.415
ZISZ/Fe	0	0.833	2.499	0.071	1.517
	1.23	-0.397	1.269	-1.159	0.287

Supplementary references

1. Wang, H., et al. Highly active deficient ternary sulfide photoanode for photoelectrochemical water splitting. *Nat. Commun.* **11**, 3078 (2020).
2. David, S., Mahadik, M. A., An, G. W., Ryu, J., Kim, H. G., Jang, J. S. Effect of directional light dependence on enhanced photoelectrochemical performance of $ZnIn_2S_4/TiO_2$ binary heterostructure photoelectrodes. *Electrochim. Acta* **276**, 223-232 (2018).
3. Xiong, Y., et al. Highly efficient photoelectrochemical water oxidation enabled by enhanced interfacial interaction in 2D/1D $In_2S_3@Bi_2S_3$ heterostructures. *J. Mater. Chem. A* **8**, 5612-5621 (2020).
4. Tian, W., Chen, C., Meng, L., Xu, W., Cao, F., Li, L. PVP treatment induced gradient oxygen doping in In_2S_3 nanosheet to boost solar water oxidation of WO_3 nanoarray photoanode. *Adv. Energy Mater.* **10**, 1903951 (2020).
5. Hou, J., et al. Atomically thin mesoporous In_2O_{3-x}/In_2S_3 lateral heterostructures enabling robust broadband-light photo-electrochemical water splitting. *Adv. Energy Mater.* **8**, 1701114 (2017).
6. Mahadik, M. A., Shinde, P. S., Cho, M., Jang, J. S. Metal oxide top layer as an interfacial promoter on a $ZnIn_2S_4/TiO_2$ heterostructure photoanode for enhanced photoelectrochemical performance. *Appl. Catal. B Environ.* **184**, 337-346 (2016).
7. Zhou, M., Liu, Z., Song, Q., Li, X., Chen, B., Liu, Z. Hybrid 0D/2D edamame shaped $ZnIn_2S_4$ photoanode modified by Co-Pi and Pt for charge management towards efficient photoelectrochemical water splitting. *Appl. Catal. B Environ.* **244**, 188-196 (2019).
8. Hassan, M. A., Waseem, A., Johar, M. A., Bagal, I. V., Ha, J.-S., Ryu, S.-W. Single-step fabrication of 3D hierarchical ZnO/ZnS heterojunction branched nanowires by MOCVD for enhanced photoelectrochemical water splitting. *J. Mater. Chem. A* **8**, 8300-8312 (2020).
9. Fu, Y., et al. Phase-modulated band alignment in CdS nanorod/SnS_x nanosheet hierarchical heterojunctions toward efficient water splitting. *Adv. Funct. Mater.* **28**, 1706785 (2018).
10. Bai, Z., Yan, X., Kang, Z., Hu, Y., Zhang, X., Zhang, Y. Photoelectrochemical performance enhancement of ZnO photoanodes from $ZnIn_2S_4$ nanosheets coating. *Nano Energy* **14**, 392-400 (2015).
11. Giri, B., et al. Balancing light absorption and charge transport in vertical SnS_2 nanoflake photoanodes with stepped layers and large intrinsic mobility. *Adv. Energy Mater.* **9**, 1901236 (2019).
12. Meng, L., et al. A plasma-triggered O-S bond and P-N junction near the surface of a SnS_2 nanosheet array to enable efficient solar water oxidation. *Angew. Chem. Int. Ed.* **58**, 16668-16675 (2019).
13. Zhang, P., Guan, B. Y., Yu, L., Lou, X. W. Facile synthesis of multi-shelled ZnS-CdS cages with enhanced photoelectrochemical performance for solar energy conversion. *Chem* **4**, 162-173 (2018).
14. Cao, S., Yan, X., Kang, Z., Liang, Q., Liao, X., Zhang, Y. Band alignment engineering for improved performance and stability of $ZnFe_2O_4$ modified CdS/ZnO nanostructured photoanode for PEC water splitting. *Nano Energy* **24**, 25-31 (2016).
15. Bhat, S. S. M., et al. Substantially enhanced photoelectrochemical performance of TiO_2 nanorods/CdS nanocrystals heterojunction photoanode decorated with MoS_2 nanosheets. *Appl. Catal. B Environ.* **259**, 118102 (2019).
